# Supplementary material for: Evaluation of scientific and technological innovation efficiency at Shandong Vocational and Technical University of international studies: A two-stage DEA model approach
Source: PLoS One. 2025 May 7;20(5):e0318020. doi: 10.1371/journal.pone.0318020 (PMC12057959; doi:10.1371/journal.pone.0318020)
Supplement: Attachment — Submitted filename: Response to Reviewers.docx [file pone.0318020.s002.docx]

Dear Editors and Reviewers:

Thank you for your letter and for the reviewers' comments concerning our manuscript entitled "Construction of Science and Technology Innovation Efficiency Evaluation System for Vocational Undergraduate Colleges" (ID:PONE-D-24-

21161). Those comments are all valuable and very helpful for revising and improving our paper, as well as the important guiding significance to our researches. We have studied comments carefully and have made correction which we hope meet with approval. Revised portion are marked with a different color in the paper. The main corrections in the paper and the responds to the reviewer's comments are as flowing:

Responds to the reviewer's comments:

Journal requirements:

When submitting your revision, we need you to address these additional requirements.

1. Please ensure that your manuscript meets PLOS ONE's style requirements, including those for file naming. The PLOS ONE style templates can be found at

https://journals.plos.org/plosone/s/file?id=wjVg/PLOSOne_formatting_sample_main_body.pdf and

https://journals.plos.org/plosone/s/file?id=ba62/PLOSOne_formatting_sample_title_authors_affiliations.pdf

Response:

We have revised the manuscript to ensure it meets PLOS ONE's formatting and style requirements. Specifically, we have made adjustments to the file naming, manuscript structure, and other formatting elements based on the guidelines provided in the PLOS ONE style templates:

1.Main Body Formatting:

We have structured the main body of the manuscript according to the template provided at PLOS ONE Formatting Sample - Main Body. This includes the correct use of headings, subheadings, and consistent formatting throughout the text.

2.Title, Authors, and Affiliations:

We have updated the title page, author names, and affiliations as per the sample template available at PLOS ONE Formatting Sample - Title, Authors, Affiliations. These changes ensure clarity and consistency in the representation of authorship and institutional details.

3.File Naming:

All files, including the manuscript, figures, and supplementary information, have been renamed according to the PLOS ONE file naming conventions to facilitate smooth submission and review.

We are confident that these revisions align our manuscript with the PLOS ONE style requirements. Thank you for your guidance, and we remain available to make any further adjustments if needed.

2. We note that your Data Availability Statement is currently as follows: [All relevant data are within the manuscript and its Supporting Information files.]

Please confirm at this time whether or not your submission contains all raw data required to replicate the results of your study. Authors must share the "minimal data set" for their submission.

PLOS defines the minimal data set to consist of the data required to replicate all study findings reported in the article, as well as related metadata and methods (https://journals.plos.org/plosone/s/data-availability#loc-minimal-data-set-definition).

For example, authors should submit the following data:

- The values behind the means, standard deviations and other measures reported;

- The values used to build graphs;

- The points extracted from images for analysis.

Authors do not need to submit their entire data set if only a portion of the data was used in the reported study.

If your submission does not contain these data, please either upload them as Supporting Information files or deposit them to a stable, public repository and provide us with the relevant URLs, DOIs, or accession numbers.

For a list of recommended repositories, please see https://journals.plos.org/plosone/s/recommended-repositories.

If there are ethical or legal restrictions on sharing a de-identified data set, please explain them in detail (e.g., data contain potentially sensitive information, data are owned by a third-party organization, etc.) and who has imposed them (e.g., an ethics committee).

Please also provide contact information for a data access committee, ethics committee, or other institutional body to which data requests may be sent. If data are owned by a third party, please indicate how others may request data access.

Response:

We have provided all the data required to meet PLOS ONE's definition of the minimal data set.

1.Values Behind Reported Measurements

We have included the values behind the reported means, standard deviations, and other statistical measures in the Supporting Information files. These data ensure transparency and allow readers to replicate the reported findings.

|  | count | mean | std | min | 25% | 50% | 75% | max |
| --- | --- | --- | --- | --- | --- | --- | --- | --- |
| X_1j_ | 10 | 1499.7 | 285.2068 | 1128 | 1259 | 1483 | 1705 | 1962 |
| X_2j_ | 10 | 3990.6 | 307.2520 | 3516 | 3738.75 | 4001 | 4221.5 | 4439 |
| X_3j_ | 10 | 721.6 | 28.4339 | 685 | 699 | 718.5 | 742.5 | 768 |
| X_4j_ | 10 | 602.2 | 29.3742 | 560 | 580 | 600 | 625.5 | 646 |
| X_5j_ | 10 | 4574 | 205.2749 | 4260 | 4435 | 4545 | 4732.5 | 4900 |
| Z_1j_ | 10 | 114.5 | 20.8127 | 85 | 96.75 | 117 | 131 | 142 |
| Z_2j_ | 10 | 96.2 | 14.7934 | 75 | 86 | 94.5 | 107.25 | 121 |
| Z_3j_ | 10 | 3740 | 1230.3568 | 2100 | 2575 | 3800 | 4750 | 5400 |
| H_1j_ | 10 | 149.7 | 15.6847 | 125 | 139 | 150.5 | 161 | 172 |
| H_2j_ | 10 | 143.8 | 17.0672 | 112 | 132.75 | 145 | 156 | 167 |
| H_3j_ | 10 | 525 | 98.3475 | 380 | 447.5 | 530 | 602.5 | 660 |
| H_4j_ | 10 | 166.8 | 20.7300 | 139 | 152.75 | 164.5 | 178.75 | 208 |
| Y_1j_ | 10 | 995 | 161.4001 | 680 | 920 | 1015 | 1110 | 1200 |
| Y_2j_ | 10 | 338 | 109.1177 | 180 | 257.5 | 335 | 402.5 | 510 |
| Y_3j_ | 10 | 221.2 | 30.1581 | 178 | 198.25 | 218.5 | 244.25 | 268 |
| Y_4j_ | 10 | 242.2 | 22.1901 | 205 | 227.25 | 245 | 257.25 | 276 |
| Y_5j_ | 10 | 304.5 | 31.5392 | 256 | 280.75 | 307.5 | 327.5 | 350 |
| Y_6j_ | 10 | -155.6 | 20.9613 | -185 | -173.25 | -153 | -140 | -126 |

2.Values Used to Build Graphs

The numerical values used to construct Figure 2 and other graphical representations have been included in the Supporting Information. These values are fully accessible to facilitate replication and verification of the results.

3.Points Extracted from Images

If applicable, we have also provided the points extracted from images or graphical analysis to ensure completeness.

All data are uploaded as Supporting Information files alongside the manuscript. If further information or clarification is required, we are happy to provide additional details. Thank you for the opportunity to ensure compliance with PLOS ONE's data availability requirements.

3. Please ensure that you include a title page within your main document. You should list all authors and all affiliations as per our author instructions and clearly indicate the corresponding author.

Response:

We have revised the manuscript to include a title page within the main document. The title page now contains the following information in accordance with the PLOS ONE author instructions:

1.Title: The full manuscript title is clearly stated.

2.Authors: All authors' full names are listed in the correct order of contribution.

3.Affiliations: Each author's institutional affiliation is provided in detail, including the institution name, department, and location.

4.Corresponding Author: The corresponding author is clearly indicated, along with their email address and institutional affiliation.

This ensures compliance with PLOS ONE's requirements. If further modifications are needed, we are happy to address them promptly. Thank you for your guidance.

Reviewer #1: Overall evaluation

This paper presents detailed data from Shandong University of Vocational and Technical International Studies for the period 2013-2022. I find the subject matter of the paper valuable and have enjoyed the scientific approach.

The tables present basic data, normalised results and evaluation results of scientific and technological innovation efficiency. It is very valuable that the author analyses a 10-year data and shows the difference with the evaluation.

I can understand the use of modified titles for your paper, but you should take into account the journal guidelines and the reader's habits of browsing the research.

The author's focus on the evaluation system and showing that it works in evaluation is also understandable and deserves respect.

However, we have come a long way in terms of access to data and the use of smart evaluation systems and the author should focus less on the evaluation system and more on what it evaluates and to what extent it explains the resulting change.

Readers and researchers will be interested in the section where the study states that from 2013 to 2022 there was a significant increase in the productivity of science and technology innovation at the university, revealing that the value of productivity increased.

I would very much like this section to be discussed in connection with the findings.

Response:

Thank you for your constructive and thoughtful feedback. We greatly appreciate your acknowledgment of the value and scientific approach of the paper. Below, we address your comments and outline the revisions we have made to improve the manuscript:

1. Title and Journal Guidelines

We understand the importance of aligning the paper's title with journal guidelines and reader habits. While we originally modified the title to emphasize the evaluation system, we recognize that readers may prefer a focus on the practical application and insights derived from the evaluation. Therefore, we will refine the title to better reflect the study's findings and implications.

2. Shift in Focus

Your comment about focusing more on what the evaluation system evaluates and the extent to which it explains the changes in productivity is well-taken.

We have expanded the Discussion section to delve deeper into the drivers of the observed increase in productivity, We discuss how these factors contributed to the productivity trends and provide evidence from the data to support these observations.

3. Linking Evaluation to Findings

We have enriched the section discussing the increase in scientific and technological productivity from 2013 to 2022 by quantifying the productivity growth and linking it to specific input and output indicators, such as the rise in the number of published papers, patents granted, and income from technology transfer, while also providing a more detailed explanation of how the evaluation system effectively captured these changes over time with a focus on its relevance and reliability, and discussing the implications of these findings for other universities and policy-making, thereby emphasizing the potential for replication and application in similar institutions.

4.Integration of Findings with Discussion

To enhance the readability and relevance of the manuscript, We have explicitly linked the findings from the evaluation system with the observed trends in productivity. This includes connecting the annual efficiency scores to the specific institutional changes that occurred during the 10-year period. We provide a comparative analysis of the university's productivity relative to regional and national benchmarks, where data is available.

5. Conclusion

We have revised the Conclusion to summarize the key findings related to productivity trends and highlight how the evaluation system contributed to uncovering these trends. This ensures that readers and researchers can easily grasp the main insights from the study.

We believe these revisions address your concerns and enhance the manuscript's focus, readability, and scientific value. Thank you again for your valuable suggestions, and we look forward to further improving the paper based on your feedback.

The Abstract

1.Research has clear objective, but it could be more specific. Mentioning the goal of improving or evaluating the efficiency of innovation activities would provide better context.

Response:

Thank you for your thoughtful comment. We agree that clarifying the specific objective of the research would improve the context and provide greater clarity for readers. In the current form, the objective of the paper focuses on evaluating the scientific and technological innovation efficiency at Shandong Vocational and Technical University of International Studies. However, explicitly stating the goal of improving and evaluating the efficiency of innovation activities would provide a clearer and more actionable context.

In the revised version of the paper, we will specify the dual objective of the research, which is to evaluate the efficiency of scientific and technological innovation (STI) activities at Shandong Vocational and Technical University of International Studies by applying a two-stage DEA (Data Envelopment Analysis) model and to identify areas for improvement in the university's STI activities.

This dual focus aims to optimize the allocation of resources, enhance innovation outcomes, and facilitate the transformation of research results into practical applications. By explicitly mentioning both the evaluation and improvement goals, we aim to make the paper's objectives clearer, while also highlighting the practical implications for institutional development.

2.Explaining why the increase in research efficiency is important and how it impacts the institution, or the field of vocational education would add value. The abstract mentions proposing policy recommendations but does not provide any details.

Response:

Thank you for your valuable feedback. We appreciate your point that the importance of increased research efficiency and its impact on the institution or the field of vocational education could be better explained, and that more details on policy recommendations are needed.

These aspects indeed add value to the paper by providing a more comprehensive understanding of the research's significance and practical implications.

To address this, we will elaborate on the importance of increasing research efficiency and its far-reaching impact on both the university and the broader field of vocational education.

Increasing research efficiency is crucial for universities, especially vocational institutions, as it directly influences their ability to innovate, enhance the quality of education, and contribute to regional and national economic development.

Efficient research activities ensure that resources—human, financial, and infrastructural—are optimally utilized, leading to improved academic output, such as more research publications, patents, and technological solutions that address real-world problems. This also enhances the university's reputation, attracting more funding, high-level researchers, and partnerships with industry.

Furthermore, improving research efficiency fosters stronger collaboration with industry by translating scientific findings into practical applications, benefiting industries and local communities through innovative products and services.

The field of vocational education also benefits from increased research efficiency in several ways.

Efficient STI activities ensure that research outputs are relevant to current industry demands, helping vocational education institutions produce a skilled workforce equipped with up-to-date knowledge and practical expertise.

Research efficiency also contributes to the creation of new knowledge, methodologies, and technologies that can be integrated into teaching programs, enhancing the quality of education and better preparing students for future careers.

Additionally, vocational universities that enhance research efficiency drive local economic innovation, particularly in high-tech and emerging industries, by providing talent, solutions, and collaborations.

To strengthen the paper further, we will explicitly outline policy recommendations based on the findings of the STI efficiency evaluation. First, policymakers should ensure that universities are provided with adequate funding and support for R&D activities.

The findings indicate that certain universities, including Shandong Vocational and Technical University of International Studies, face resource constraints that hinder their ability to optimize research outputs.

A targeted investment in human capital, such as research staff, and technological infrastructure is essential.

Second, government policies should promote stronger partnerships between universities and industries, as enhanced collaboration with enterprises, particularly high-tech firms, can drive more efficient commercialization of research outputs.

Third, the government should implement region-specific policies that address the unique challenges faced by universities in different areas, focusing on improving the local innovation ecosystem, strengthening links with local industries, and providing additional resources to underperforming regions.

Finally, policies aimed at attracting and retaining top-tier researchers and faculty members are critical, as universities with strong research teams perform better in innovation.

In conclusion, the university's improved research efficiency and its broader implications for vocational education highlight the need for a detailed discussion on policy recommendations. These strategies, supported by the evidence presented, will ensure a comprehensive understanding of the research's significance and practical applications. We will incorporate these points into the revised manuscript to provide a more robust and data-driven analysis.

3.Including a brief mention of the type of recommendations (e.g., funding allocation, curriculum changes) would make the abstract more informative and appealing to readers.

The abstract has enough space to add more detail about the policy recommendations and the significance of the findings without exceeding the limit.

Response:

Thank you for your valuable suggestion. We agree that including a brief mention of the types of recommendations—such as funding allocation and curriculum changes—would make the abstract more informative and engaging for readers.

To enhance the clarity and appeal of the abstract, we will revise it to include specific examples of the policy recommendations derived from the study's findings.

Materials and Methods

4.The Materials and Methods section should provide enough detail to allow suitably skilled investigators to fully replicate your study. But this part of the article is hard to find and hard to follow.

Response:

Thank you for your insightful feedback. We understand the importance of a clearly structured Materials and Methods section to ensure that the study can be fully replicated by other researchers. We acknowledge that this section was not sufficiently clear and well-defined in its current form. To address this, we will revise the article by creating a dedicated and well-structured Materials and Methods section, ensuring that all aspects of the research design, data collection, and model specifications are presented in a transparent and replicable manner.

1. Data Collection:

This study focuses on evaluating the scientific and technological innovation (STI) efficiency of Shandong Vocational and Technical University of International Studies (SVTUIS). The data used in the study spans from 2013 to 2022 and includes both input and output indicators for STI efficiency evaluation. The data were obtained from internal university records and publicly available sources.

1.1Input indicators: R&D full-time personnel, R&D expenditure, and other funding resources.

1.2 Output indicators: The number of academic papers published, patents granted, technology transfer income, and research awards.

1.3 Additional inputs for results transformation stage: New product R&D costs, new researchers, and related funding for commercialization.

2. Model Selection and Justification

The study employs a two-stage Data Envelopment Analysis (DEA) model to evaluate STI efficiency at SVTUIS. The two stages are: (1) knowledge innovation stage, which measures the efficiency of transforming inputs (research resources) into knowledge outputs (e.g., publications, patents), and (2) results transformation stage, which assesses how well the knowledge outputs are converted into economic and academic value (e.g., technology transfer, patents income).

2.1 DEA Model Framework: A shared-input DEA model is used, where additional new inputs are incorporated in the second stage. The model is set to evaluate the efficiency of each year from 2013 to 2022 as a Decision-Making Unit (DMU). The normalization of data follows a scaling process based on 2022 values, using the maximum value (1.1 times of 2022 values) to normalize each indicator.

3. Data Preprocessing and Normalization

Before applying the DEA model, the raw data were normalized to ensure comparability across different years. For the normalization process, the values from 2022 were used as the reference year, multiplying these values by a factor of 1.1 to ensure that the data across all years fall within a common scale.

4. DEA Model Application

The two-stage DEA model is implemented using the R software (version 3.6.1) and its DEA package. Each year (from 2013 to 2022) is treated as a separate DMU. The first stage measures the efficiency of transforming research inputs (R&D personnel, expenditure) into scientific knowledge outputs (publications, patents). The second stage evaluates how effectively these knowledge outputs are transformed into economic and academic value, using metrics like patent commercialization income and research awards.

5.Performance Evaluation and Statistical Methods

Efficiency scores for each year are calculated for both stages. Statistical tools are used to analyze the dynamic evolution of STI efficiency, including the calculation of mean efficiency scores across the entire evaluation period, as well as identifying trends of efficiency improvements or declines. In addition, sensitivity analysis is conducted to assess the robustness of the DEA results under different input/output configurations.

We will revise the manuscript to ensure that the above section is clearly presented, structured, and adequately detailed to facilitate replication. Additionally, we will ensure that the methodologies used, the rationale for choosing the model, and all data sources are transparent and comprehensible to other researchers. This revision will help ensure that the Materials and Methods section fully meets academic standards and enables other investigators to replicate the study. Thank you for pointing this out.

Results, Discussion, Conclusions

5.These sections may all be separate, or may be combined to create a mixed Results/Discussion section (commonly labeled "Results and Discussion") or a mixed Discussion/Conclusions section (commonly labeled "Discussion").

These sections may be further divided into subsections, each with a concise subheading, as appropriate. These sections have no word limit, but the language should be clear and concise.

Response:

Thank you for your thoughtful comment. We acknowledge that the Results, Discussion, and Conclusions sections of the manuscript could benefit from better clarity and structure. To improve the overall readability and alignment with academic standards, we will separate and organize these sections more effectively, following the suggested guidelines.

1.Results

This section will present the findings derived from the two-stage DEA model. The results will be divided into two subsections to present the efficiency scores for each stage, as well as the overall analysis of efficiency trends from 2013 to 2022.

1.1.Efficiency Scores for Knowledge Innovation Stage

The first stage of the DEA model evaluates the efficiency of transforming inputs (e.g., R&D personnel, financial resources) into scientific knowledge outputs (e.g., publications, patents).

The results show an overall trend of increasing efficiency in knowledge creation, as reflected in the steady rise in the number of academic papers and patents published. Specific figures for each year will be presented in this subsection, alongside a discussion of the significant changes over time.

1.2. Efficiency Scores for Results Transformation Stage

The second stage assesses how effectively the outputs of knowledge innovation (publications and patents) are transformed into economic and academic value (e.g., income from technology transfer, research awards). This subsection will highlight the efficiency trends in the commercialization of research outputs, including the increased income from patents and technology transfers.

1.3. Overall Efficiency Analysis

This subsection will combine the efficiency results of both stages and provide a comprehensive analysis of the trends from 2013 to 2022. We will discuss the relationship between the two stages and how improvements in knowledge innovation lead to enhanced results transformation efficiency.

1.4. Sensitivity and Robustness Analysis

To ensure the reliability of our results, we will also present the findings of the sensitivity analysis, which tests the robustness of our results under various assumptions and configurations. This will provide a deeper understanding of the stability of the efficiency trends over time.

2.Discussion

The Discussion section will interpret the results in the broader context of scientific and technological innovation in universities, particularly in vocational and technical institutions.

2.1. Implications of Results

This subsection will explore the implications of the results for improving STI efficiency in vocational universities. We will compare our findings with previous studies on university research efficiency, such as those by Chen et al. (2014) and Abramo et al. (2013), and discuss how different input and output factors influence STI efficiency in vocational institutions. For example, we will address the role of faculty expertise, funding allocation, and the university's strategic focus on innovation in driving improvements in research productivity and commercialization.

2.2. Factors Influencing Efficiency

Here, we will delve deeper into the factors that contributed to the observed improvements in research efficiency. For example, the introduction of high-level research talents, increased R&D investment, and greater collaboration with industry could have significantly influenced the efficiency scores. We will also examine whether the university's institutional strategies, such as a focus on applied research or specific technological domains, played a role in enhancing its STI outcomes.

2.3. Comparison with Other Universities

In this subsection, we will briefly compare the results from Shandong Vocational and Technical University of International Studies with similar universities in China or internationally. This will help to contextualize the findings and highlight areas where the university could improve further, as well as best practices that may be transferable to other institutions.

2.4. Limitations of the Study

We will clearly state the limitations of the current study, including potential data gaps, the focus on a single university, and the challenges of generalizing findings to other institutions.

Additionally, we will discuss the constraints imposed by the use of DEA models, such as the assumption of linear relationships between inputs and outputs, and the exclusion of qualitative factors that may also influence STI efficiency.

3.Conclusions

The Conclusions section will summarize the main findings of the study and highlight the broader implications for policy and practice.

3.1. Summary of Key Findings

We will summarize the main results of the DEA analysis, including the overall efficiency trends in STI activities at Shandong Vocational and Technical University of International Studies. The summary will focus on the efficiency improvements observed in both knowledge innovation and results transformation stages.

3.2. Policy Implications and Recommendations

Based on the results, we will propose several actionable recommendations for enhancing the efficiency of STI activities at vocational universities. These may include suggestions for increasing investment in R&D, fostering partnerships with industry, improving the management of research resources, and strengthening the commercialization of research outputs.

3.3. Future Research Directions

Finally, we will outline directions for future research in this area. This could include expanding the study to include more universities, investigating the impact of specific policy interventions, or exploring the use of alternative evaluation models (e.g., Malmquist Index, stochastic frontier analysis).

We will revise the manuscript accordingly, separating and expanding the Results, Discussion, and Conclusions sections with appropriate subsections, as outlined above. The revised sections will be more concise, focused, and supported by the data and references to relevant literature.

Materials and Methods

6. Study Design: Please describe the overall design of the study, including the objectives and hypotheses.

Response:

Thank you for your insightful comment. We will revise the Materials and Methods section to provide a clear and comprehensive description of the overall study design.

1.Study Design

This study aims to assess the efficiency of scientific and technological innovation (STI) activities in Shandong Vocational and Technical University of International Studies over a ten-year period, from 2013 to 2022. The primary objective is to evaluate the university's STI efficiency, considering both the knowledge innovation and results transformation stages of the innovation process. This will allow us to analyze how the university's research outputs (publications, patents, etc.) have contributed to its overall scientific and technological advancement.

The study employs a two-stage Data Envelopment Analysis (DEA) model, which is a well-established method for evaluating the efficiency of decision-making units (DMUs) in multi-input, multi-output settings. Specifically, the study evaluates the efficiency of STI activities at the university using input indicators such as R&D personnel and expenditures, and output indicators like academic papers, patents, and income from technology transfer. The two-stage approach allows for the separation of the innovation process into two distinct phases: knowledge innovation and results transformation, both of which contribute to the overall efficiency of the institution.

2. Objectives of the Study

2.1To evaluate the efficiency of scientific and technological innovation activities at Shandong Vocational and Technical University of International Studies using a two-stage DEA model.

2.2To analyze the efficiency trends from 2013 to 2022, with a focus on both the knowledge innovation and results transformation stages.

2.3To identify the key factors that influence STI efficiency, such as R&D investment, research talent, and collaboration with industry.

2.4To assess the implications of STI efficiency for improving the university's research productivity and its contribution to regional economic development.

3.Hypotheses

H1: The efficiency of scientific and technological innovation at Shandong Vocational and Technical University of International Studies has significantly improved over the past decade.

H2: The efficiency of the knowledge innovation stage (input-to-output) is positively correlated with the efficiency of the results transformation stage (output-to-economic value).

H3: Increasing R&D investment and the introduction of high-level research talents will have a positive impact on the STI efficiency of the university.

H4: The commercialization of research outputs, such as patents and technology transfers, significantly influences the overall STI efficiency.

7. Data Collection: Please give detail the data sources, including the period (2013-2022) and the specific data collected from Shandong Vocational and Technical University of International Studies.

Response:

Thank you for your valuable suggestion. We will revise the Data Collection section to provide a more detailed description of the data sources and the specific data collected from Shandong Vocational and Technical University of International Studies for the period 2013-2022. This section will clarify the sources of data, the types of data collected, and the methodology used for data compilation.

1.Data Collection

This study utilizes data from Shandong Vocational and Technical University of International Studies (SVTUIS) to evaluate the efficiency of scientific and technological innovation (STI) activities over the period 2013-2022. The data were collected from various administrative and research departments within the university, including the Research Office, Financial Department, and the Office of Academic Affairs. The collected data pertain to both input (resources allocated to R&D activities) and output (research results and their economic value) indicators, which were used to assess STI efficiency.

2. Period of Data Collection

The study covers a ten-year period from 2013 to 2022. This period was chosen to provide a comprehensive overview of the university's STI activities and to observe trends over time. The data for each year were collected from official university records, ensuring consistency and accuracy in the dataset.

3.Specific Data Collected

The data collected for the evaluation of STI efficiency include the following:

3.1 Input Indicators

3.1.1 R&D Full-time Personnel (X_1j_)

The number of full-time personnel involved in research and development, including researchers, staff working on applied research, and individuals contributing to technology service. This data was sourced from the university's HR department.

3.1.2 R&D Expenditure (X_2j_)

The annual internal expenditure dedicated to scientific research activities, including funds allocated to various research projects. The data was obtained from the university's Financial Department.

3.1.3 Laboratory Base Inputs (X_3j_)

The share of resources allocated to laboratory infrastructure and research facilities, obtained from the university's facilities management department.

3.1.4 R&D FTE Conversion (X_4j_)

The full-time equivalent (FTE) conversion of R&D personnel. This represents the total research labor force, expressed in person-years. The data was collected from the university's HR and research departments.

3.1.5 Gross Salary of Researchers (X_5j_)

The total salary expenses for research personnel, reflecting the financial commitment to human resources in research. This data was provided by the university's payroll records.

3.2 Output Indicators

3.2.1 Academic Papers Published (Z_1j_)

The total number of academic papers published by university researchers in indexed journals. This data was collected from the university's research output database, including papers published in CSSCI (Chinese Social Sciences Citation Index) journals

3.2.2 Patents Granted (Z_2j_)

The number of patents granted based on research outcomes. Data on patents granted were obtained from the university's Intellectual Property Office.

3.2.3 Graduates (Z_3j_)

The number of students graduating from programs directly related to R&D or science and technology, as a proxy for the human resource output of the university. This data was provided by the university's student affairs office.

3.3 Additional Input Indicators (for Stage 2: Results Transformation)

3.3.1 New Technology R&D and Transformation Costs (H_1j_)

The expenditure on R&D specifically dedicated to the development and transformation of new technologies and products. The data was obtained from financial records related to technology commercialization efforts.

3.3.2 New Full-time Researchers (H_2j_)

The number of new full-time researchers hired in areas directly contributing to applied R&D and technology transformation. Data was collected from the HR department.

3.3.3 Total Payroll for New Personnel (H_3j_)

The total salary expenses for newly hired researchers in applied R&D. This data was obtained from payroll records.

3.3.4 Other Costs to be Increased or Shared (H_4j_)The costs associated with collaboration and the sharing of research resources between departments or with external partners. The data was obtained from the university's financial office.

3.4 Final Output Indicators

3.4.1 Academic Papers Published (Y_1j_)

The number of academic papers published by the university in high-impact journals, with data collected from the university's research output database.

3.4.2 Patents Obtained (Y_2j_)

The number of patents obtained by university researchers, with data sourced from the Intellectual Property Office.

3.4.3 Scientific and Technical Publications Points (Y_3j_)

A points system assigned to publications based on their impact and prestige, which reflects the quality of research output. Data on publication points were provided by the university's academic affairs office.

3.4.4 Income from Technology Transfer (Y_4j_)

The revenue generated from the transfer of technologies or patents to external organizations or industries. This data was collected from the university's commercialization and licensing office.

3.4.5 Research Awards (Y_5j_)

Points for scientific research awards won by the university or its researchers, reflecting the recognition of the university's research output. Data on research awards were provided by the university's academic office.

3.4.6 Research Waste or Loss (Y_6j_)

The value of research resources that were unproductive or resulted in negative outcomes, including research waste or loss. This data was collected from the financial department and the research office.

4.Data Processing

Once the data were collected, they were normalized to account for differences in scale between the input and output indicators. As the data on research waste (Y_6j_) had negative values, they were treated as undesirable outputs, and a specific transformation was applied to make them positive for inclusion in the analysis. This normalization process ensures comparability and avoids biases in the DEA evaluation.

5.Data Sources

5.1Shandong Vocational and Technical University of International Studies Official Reports (2013-2022)

5.2 University's Research Office (Academic papers, Patents)

5.3University's Financial Department (Expenditure data, Payroll records)

5.4 University's HR Department (Personnel data)

5.5University's Intellectual Property Office (Patent and Technology Transfer Data)

5.6 University's Student Affairs Office (Graduation data)

This data collection process ensures that the study is based on reliable, verifiable information, and allows for an accurate evaluation of the university's STI efficiency.

8. Indicators Selection: Please list and describe the input and output indicators used in the study, such as R&D full-time personnel, R&D expenditure, number of published scientific writings, and patents granted.

Response:

Thank you for the suggestion to provide a detailed description of the input and output indicators used in the study. Below is the revised section on Indicators Selection, which lists and describes the specific input and output indicators utilized to evaluate the scientific and technological innovation (STI) efficiency of Shandong Vocational and Technical University of International Studies (SVTUIS) from 2013 to 2022.

In this study, a comprehensive set of input and output indicators was selected to evaluate the efficiency of scientific and technological innovation (STI) activities at Shandong Vocational and Technical University of International Studies (SVTUIS). The indicators were chosen based on their relevance to the innovation process, their ability to reflect the effectiveness of research activities, and their availability for the study period (2013-2022). The indicators used in this study are organized into input indicators, intermediate output indicators, and final output indicators.

1.Input Indicators

1.1 R&D Full-time Personnel (X_1j_)

This indicator measures the total number of full-time personnel involved in scientific research, technology development, and technological services at the university. It includes researchers, R&D staff, and personnel dedicated to research administration and project management. This input is crucial because the human resources in R&D activities are a direct determinant of the capacity for scientific innovation and output.

1.2 R&D Expenditure (X_2j_)

This indicator represents the total annual internal expenditure dedicated to R&D activities. R&D expenditure includes the funding for research projects, laboratory operations, and technological services. It is an essential measure of the financial investment made in scientific and technological innovation.

1.3 Laboratory Base Inputs (X_3j_)

This indicator refers to the share of resources allocated to research infrastructure, including the maintenance and upgrading of laboratories, research equipment, and technical facilities. A strong laboratory base is vital for fostering innovation and supporting research projects.

1.4 R&D FTE (Full-Time Equivalent) Conversion (X_4j_)

This indicator reflects the total amount of labor devoted to R&D activities, measured in full-time equivalent (FTE) years. It is calculated by converting the time spent by all researchers into person-years. This input is essential to assess the extent of human capital applied to scientific research.

1.5 Gross Salary of Researchers (X_5j_)

This indicator represents the total salary expenses for R&D personnel at the university. It provides insight into the university's investment in human capital for research and innovation. Higher salary expenditures are often associated with attracting high-caliber researchers and fostering a competitive research environment.

2. Intermediate Output Indicators

2.1 Number of Academic Papers Published (Z_1j_)

This indicator measures the total number of academic papers published by university researchers in indexed journals. Publications are a key outcome of scientific research and indicate the university's contribution to advancing knowledge in various fields. Papers published in journals such as those indexed in the Chinese Social Sciences Citation Index (CSSCI) are given particular importance in this study.

2.2 Number of Patents Granted (Z_2j_)

This indicator tracks the number of patents granted to university researchers, representing the commercialization potential of research outputs. Patents are a key measure of the university's capacity to translate research into tangible innovations with potential economic value.

2.3 Number of Graduates (Z_3j_)

This indicator measures the number of students graduating from programs directly related to R&D or science and technology. Graduates serve as a measure of the university's ability to cultivate a skilled workforce that can contribute to the development of new technologies and innovations.

3.Final Output Indicators (Results Transformation Stage)

3.1 Conversion Score for Academic Paper Publications (Y_1j_)

This indicator measures the academic value generated by the publication of scientific papers. It takes into account the quality, impact, and citation frequency of the published papers. Higher scores are assigned to papers published in higher-impact journals or those that are widely cited by the academic community.

3.2 Number of Patents Obtained (Y_2j_)

This final output indicator measures the number of patents granted to the university's R&D teams during the year. Patents are an important metric for the successful transformation of research into practical, commercially viable technologies.

3.3 Points for Scientific and Technical Publications (Y_3j_)

This indicator measures the impact of scientific and technical publications based on their contribution to advancing scientific knowledge and technology. The scoring system accounts for the journal's quality and the citation rate of published works.

3.4 Income from Technology Transfer (Y_4j_)

This indicator measures the financial income generated from the transfer of technology, including royalties and licensing fees. It reflects the economic impact of the university's R&D outputs and their successful commercialization.

3.5 Research Awards (Y_5j_)

This indicator tracks the number of research awards received by university researchers. It includes national and provincial research awards that acknowledge the scientific and technological achievements of the university. Higher awards indicate more significant contributions to scientific and technological progress.

3.6 Research Waste or Loss (Y_6j_)

This indicator measures the financial loss or waste incurred in R&D activities, including failed research projects and unutilized research outputs. Negative values in this category reflect inefficiencies in the R&D process, and the goal is to minimize this output. In the model, research waste was treated as an undesirable variable, with appropriate adjustments made for its negative values (as detailed in the methodology section).

These indicators were selected to cover all aspects of the scientific and technological innovation process, from human and financial inputs to the outputs in terms of academic contributions and commercialization of research. The use of both input and output indicators ensures a comprehensive evaluation of the university's STI efficiency and helps identify areas for improvement in resource allocation, research performance, and technology commercialization.

9. Model Construction: Please explain the shared-input-type correlation DEA model and the two-stage network DEA model. Describe how intermediate output reinvestment, initial input resource sharing, and new inputs were factored into the model.

Response:

Thank you for the request for clarification regarding the model construction. Below is a detailed explanation of the shared-input-type correlation DEA model and the two-stage network DEA model, including how intermediate output reinvestment, initial input resource sharing, and new inputs were factored into the model.

1.Model Construction

This study utilizes two advanced modeling approaches to evaluate the efficiency of scientific and technological innovation (STI) activities in Shandong Vocational and Technical University of International Studies (SVTUIS). The two models applied are the shared-input-type correlation DEA model and the two-stage network DEA model. These models are particularly suitable for capturing the multi-stage nature of STI activities, which include knowledge innovation and results transformation.

1.1.Shared-Input-Type Correlation DEA Model

The shared-input-type correlation DEA model is designed to handle situations where inputs are shared across multiple stages of production or where certain resources are used both at the initial stage (knowledge innovation) and the subsequent stage (results transformation). In the context of STI, the inputs in the knowledge innovation stage (such as R&D personnel and funding) are partially used in the results transformation stage (such as technological applications and commercialization).

1.1.1 Key Features of the Shared-Input-Type DEA Model

Input Sharing: Some inputs are shared between stages (e.g., R&D funding, research staff) and thus need to be allocated appropriately between the two stages to prevent double-counting.

Intermediate Output Reinvestment: The outputs produced in the first stage (knowledge innovation) become inputs in the second stage (results transformation). For example, academic papers and patents generated in the knowledge innovation stage may be reinvested into the transformation stage, where they are commercialized or applied in industry.

Resource Allocation: The model requires careful allocation of shared inputs and outputs to each stage to accurately assess efficiency. This allocation involves assigning specific proportions of input resources to each stage based on the contribution they make to the respective output.

In practice, the model calculates efficiency by considering the relationship between inputs and outputs across both stages. For the initial input resources (such as R&D expenditure and personnel), the DEA model calculates the efficiency of each stage separately while accounting for shared inputs and their impact on both outputs.

1.2. Two-Stage Network DEA Model

The two-stage network DEA model is an extension of the standard DEA model that explicitly accounts for the sequential, multi-stage nature of the production process. In this case, the STI process is decomposed into two stages.

Stage 1 – Knowledge Innovation: This stage involves the creation of new knowledge through R&D activities, such as publications and patents.

Stage 2 – Results Transformation: This stage focuses on the application of research results, including commercialization, technology transfer, and economic value generation from patents and publications.

1.2.1 Key Features of the Two-Stage Network DEA Model

Stage 1: In this stage, the inputs (such as R&D personnel and expenditure) contribute to outputs (e.g., academic papers and patents) that serve as the intermediate outputs for Stage.

Stage 2: The intermediate outputs from Stage 1 (e.g., academic papers and patents) become inputs in Stage 2, where they are transformed into economic and technological value, such as income from technology transfer and commercialization.

This model allows for the measurement of the efficiency of each stage separately, while also providing a comprehensive assessment of the overall STI efficiency by integrating the two stages. The intermediate outputs (like publications and patents) are reinvested in Stage 2 to reflect the dynamic process of innovation. This reinvestment is captured in the model, showing how outputs from the first stage drive the efficiency of the second stage.

2. Intermediate Output Reinvestment, Initial Input Resource Sharing, and New Inputs in the Model

2.1 Intermediate Output Reinvestment

In the two-stage model, the outputs from the first stage (e.g., academic papers and patents) are reinvested into the second stage. The reinvestment is considered an input to Stage 2, and its effect on the efficiency of results transformation is evaluated. This reinvestment is critical because it reflects the innovation value chain, where scientific outputs contribute to economic or technological applications.

2.2 Initial Input Resource Sharing

The shared-input-type DEA model captures how certain input resources (such as R&D personnel and funding) are used across both stages. In the model, shared inputs are allocated proportionally to the two stages to avoid double-counting and to ensure that the contribution of these inputs to both knowledge creation and results transformation is accurately reflected.

2.3 New Inputs in Stage 2

The model also considers new inputs required for the results transformation stage. For example, investments in technology development and commercialization (such as new product development or funding for patent commercialization) are incorporated as new inputs in Stage 2. These new inputs are necessary for the successful transformation of knowledge into tangible outputs like technological products and services.

10. Data Analysis: Please give detail the analysis and explain the normalization process for the evaluation indicators.

Response:

Thank you for your insightful comment regarding the data normalization process and the selection of the reference maximum value for the evaluation indicators.

We acknowledge the importance of providing a clear explanation of this aspect to ensure transparency and replicability in our study. Below is our detailed response and an improved integration of this explanation into the manuscript.

In this study, the normalization process was carefully designed to ensure consistency and comparability across the evaluation period of 2013–2022. The reference maximum value for each indicator was set as 1.1 times the value of the indicator in 2022. For the input indicator (R&D Full-time Personnel) (X_1j_), the actual value in 2022 is 1962, and the maximum reference value is calculated as 1.1×1962=2158.2. Using this maximum value, the normalized value for each year was calculated using the formula:

For example, for the year 2013, where the actual value for X1j is 1128, the normalized value is calculated as:

This normalization approach was applied to all years (2013–2022) for the indicator X1j, ensuring that all normalized values fall between 0 and 1.

By choosing 1.1 times the 2022 value as the maximum reference, the normalization reflects potential future growth in STI activities, aligning with the dynamic and forward-looking nature of the evaluation framework.

This approach avoids the influence of outliers by basing normalization on a representative value from a recent year with comprehensive data availability and ensures consistency across all years and indicators, maintaining uniformity in the scale of the data.

The decision to use 1.1 times the 2022 value rather than the 2013 value (or another fixed year) was guided by the goal of aligning the evaluation with the most recent data, capturing the current state of STI activities more accurately.

By scaling the data using this multiplier, the normalization process accommodates potential growth while maintaining consistency with other indicators. The revised normalization process ensures comparability and enhances the robustness of the DEA model results.

We appreciate your careful review and feedback, which has allowed us to identify and correct this important detail. The revised calculation and explanation have been incorporated into the manuscript to ensure accuracy and clarity in the normalization process.

11. The manuscript does not have a clearly labeled "Results" or "Results and Discussion" section. However, the results and their discussion are embedded within the "Case Studies and Discussions" section.

Response:

Thank you for your observation. We appreciate your feedback regarding the structure of the manuscript, particularly in relation to the presentation of the "Results" and "Discussion" sections.

We understand that it is standard in academic writing to clearly distinguish between the Results and Discussion sections to enhance readability and clarity. In our current manuscript, the results and their analysis are indeed presented under the "Case Studies and Discussions" section, which may have caused some confusion regarding the structure of the paper.

We recognize the need to separate the Results and Discussion sections to improve the organization and clarity of the paper. Therefore, in the revised version of the manuscript, we will explicitly create a Results section, followed by a Discussion section.

The Results section will focus on presenting the findings of the DEA model, including the efficiency scores for each year, the efficiency trends observed across the years, and the specific results for each stage (knowledge innovation and results transformation).

The Discussion section will interpret these results, compare them with relevant studies, and provide insights into the implications of the findings.

By adding a separate Results section and improving the Discussion section, we aim to make the structure of the manuscript more consistent with academic conventions, allowing for clearer communication of the study's findings and interpretations.

Reviewer #2

1.The authors have serious problems with references as many mistakes occur. The references must be profoundly revised. Similarly, there are many missing spaces or extra/double spaces in the text. Moreover, please avoid using very long paragraphs within the text.

Response:

Thank you for pointing out the issues related to references, spacing, and paragraph structure in our manuscript. To address these concerns, we have thoroughly revised the text, making the following adjustments.

All references have been carefully reviewed and corrected to ensure accuracy and consistency with the journal's citation style.

Missing information, such as journal names, volume numbers, and page ranges, has been added where applicable, and formatting inconsistencies, including punctuation and alignment with the journal's guidelines, have been rectified.

Additionally, the entire manuscript has been checked for missing spaces, extra spaces, and double spaces, and these errors have been corrected to enhance readability and align with professional formatting standards.

Regarding paragraph structure, long paragraphs have been identified and split into shorter, concise sections to improve clarity and readability, with each paragraph now focusing on a single idea or point, ensuring that the manuscript adheres to academic writing conventions.

We are confident that these revisions have addressed the issues you highlighted, and we sincerely appreciate your valuable feedback.

2.Try to keep all paragraphs within 10-12 lines for better readiness. Please revise the complete article or use a proofreading service.

Response:

Thank you for your feedback regarding paragraph length and readability. We have carefully revised the manuscript to address these concerns by taking the following actions. All paragraphs have been reviewed and restructured to ensure conciseness, adhering to the recommended range of 10-12 lines. Longer paragraphs were split into smaller sections, each focusing on a single point or idea, thereby improving readability and aligning with academic writing best practices. The entire manuscript has been thoroughly proofread to eliminate grammatical errors, typos, and inconsistencies, with special attention given to sentence structure and flow to enhance clarity and maintain a professional tone throughout. Logical transitions between paragraphs and sections have been refined to ensure smooth progression of ideas, while redundant phrases and overly complex sentences were simplified to make the content more accessible to a broader audience.

We are confident that these revisions have significantly improved the manuscript's readability and alignment with your recommendations. Thank you for your valuable suggestions, and we remain open to further adjustments if necessary.

Introduction

3.- Please add a reference related to the 18th CPC National Congress in the first paragraph.

Response:

Thank you for highlighting the need to include a reference related to the 18th CPC National Congress in the first paragraph. We have carefully addressed this comment by adding a relevant citation to provide proper context and support for the statement.

4.- "…modernization country, and to accelerate…" Split the sentence into two here.

Response:

Thank you for pointing out the need to split the sentence for improved clarity. We have revised the sentence as suggested, splitting it into two distinct parts.

We appreciate your valuable feedback and remain open to any further recommendations.

5.- "In December 2020, the Ministry of Education convened…" – reference is missing. The same in "In May 2019, the Ministry of Education announced the first 15 pilot…"

Response:

Thank you for noting the missing references. The references have been formatted according to the journal's guidelines and integrated into the text. We believe these additions enhance the credibility and completeness of the manuscript. Thank you for your valuable feedback.

6.- Please rearrange chronologically the Introduction. The authors are jumping from September 2020, to December 2020, then to May 2019.

Response:

Thank you for pointing out the need to rearrange the timeline in the Introduction for better chronological flow. We have revised the section to ensure the events are presented in chronological order, starting with May 2019, followed by September 2020, and concluding with December 2020.

This restructuring improves the logical progression of the narrative and enhances the clarity and coherence of the manuscript. We appreciate your suggestion and are confident this revision has addressed the issue. Thank you!

7.- "Scientific and technological innovation activities of colleges and universities…" – reference(s) is missing in this part. Moreover, you should add a figure to display this process.

Response:

Thank you for your feedback. We have added appropriate references to support the statement, "Scientific and technological innovation activities of colleges and universities...", ensuring the section is well-supported by credible sources.

Additionally, we have included a figure to visually display the described process of scientific and technological innovation activities. This figure enhances the clarity of the explanation and provides a clear illustration for readers to better understand the process.

We appreciate your valuable suggestions and believe these updates improve the quality and comprehensiveness of the manuscript. Thank you!

8.- "Although the overall development level of scientific and technological innovation in colleges and universities in China has been significantly improved…" – how? Please, be more specific and provide some statistics, figures, etc., and support your statement with references.

Response:

Thank you for pointing out the need for specificity in the statement, "Although the overall development level of scientific and technological innovation in colleges and universities in China has been significantly improved." We have addressed your comment as follows. The statement has been expanded to include concrete data and examples illustrating improvements in scientific and technological innovation. Specifically, we now highlight the increase in the number of research papers published by Chinese universities in international journals, as reflected in global citation databases, the significant rise in patents granted to universities based on data from the National Intellectual Property Administration, and the growth in R&D expenditure in higher education institutions over the past decade, supported by relevant government statistics. These updates provide a more robust foundation for the statement and address the need for clarity and evidence. Thank you for your constructive feedback, and we believe the revised manuscript effectively reflects these improvements.

9.- Further, the same, "there are still problems such as low utilization of innovation resources and unbalanced regional development" – references are missing.

Response:

Thank you for pointing out the need to cite references to support the statement that “problems such as low innovation resource utilization and unbalanced regional development still exist”. We have addressed this issue by adding relevant references from authoritative sources. We appreciate your valuable suggestions and believe that this revision will improve the manuscript.

10.- "Although the overall development level of scientific…" – this sentence has 13 lines! Please, revise this sentence and divide it into several shorter ones to improve the readiness and flow.

Response:

Thank you for pointing out the need to revise and divide the sentence, "Although the overall development level of scientific and technological innovation in colleges and universities…", to improve readability and flow. We have carefully restructured this sentence into several shorter, concise sentences. This adjustment enhances clarity and ensures the text is easier to follow. Each sentence now focuses on a specific point, improving the overall narrative flow and alignment with academic writing standards. We appreciate your suggestion and are confident this revision has addressed the issue effectively. Thank you!

11.- "The main reasons for the inefficiency of innovation are clarified." – what does it mean? Where?

Response:

Thank you for pointing out the ambiguity of the statement, "The main reasons for the inefficiency of innovation are clarified." Upon review, we found the statement to be unnecessary and lacking specificity. In the revised manuscript, this sentence has been removed to enhance the precision and focus of the text.

We appreciate your feedback, which has helped to improve the clarity and quality of the manuscript. Thank you!

12.-Research on the influencing factors of scientific and technological innovation efficiency in universities

This chapter is only one paragraph! Divide the text into several shorter and logical paragraphs.

Response:

Thank you for pointing out the need to improve the structure of the chapter, "Research on the influencing factors of scientific and technological innovation efficiency in universities." We appreciate your suggestion and are confident that the revised version addresses your concerns effectively. Thank you!

13.-Research on the evaluation of scientific and technological innovation efficiency in universities

"…technological innovation efficiency, respectively.DIVIDE THE PARAGRAPH HERE The third category compares…".

- It is not necessary to explain all forms of the DEA methodology used in the STI evaluation (CCR, BCC, two/three stages, MI and WA models.

It is enough saying that the non-parametric approach based on the DEA methodology is used, and continue directly: "In this case, Shen and Gong (2013) used…"

Response:

Thank you for your comments on the section "Research on the evaluation of scientific and technological innovation efficiency in universities." We have implemented the suggested changes as follows.

To improve readability and logical flow, the paragraph has been divided at "…technological innovation efficiency, respectively. The third category compares…" This adjustment helps create a clearer structure for the discussion.

Additionally, we have removed the detailed explanation of various DEA models (CCR, BCC, two/three stages, MI, and WA) as per your suggestion.

Instead, we have briefly stated that a non-parametric approach based on DEA methodology was used, and continued directly with the specific application by Shen and Gong (2013).

These revisions streamline the text and ensure a concise, focused discussion, aligning with your recommendations. Thank you for your valuable input!

Selection of indicators

14.- In table 1, please better divide the inputs, intermediates and outputs, use a simple line between each category. Right now one can think, for example, that the intermediates begin on the line next to Intermediate output indicators. The same with the Additional input indicators.

Response:

Thank you for highlighting the need for better visual organization in Table 1. We have revised the table to include simple lines separating the inputs, intermediate outputs, and outputs, making it clear where each category begins and ends.

This adjustment ensures the table is more reader-friendly and minimizes any potential confusion about the categorization of indicators. We appreciate your feedback and are confident the revised table meets the expectations for clarity and presentation. Thank you!

15.- The authors mention that "…the result transformation stage include part of the initial input resources". – Which of the initial inputs are later partially used? It is not clear from Table 1.

Response:

Thank you for your comment. In the original manuscript, we mention that "the result transformation stage includes part of the initial input resources."

Initial Inputs: These refer to the resources allocated at the early stage of scientific and technological innovation, such as R&D expenditure, R&D personnel, etc. In Table 1, these are categorized as Input Indicators (e.g., X_1j_, X_2j_, X_3j_, X_4j_, X_5j_).

Intermediate Outputs: These resources are partially utilized in the second stage (the result transformation stage), which involves converting knowledge and ideas into tangible outcomes (e.g., patents, published papers, etc.). This transformation utilizes resources like R&D personnel and R&D expenditure, but in a way that is integrated into the generation of results (e.g., Y_1j_, Y_2j_, Y_3j_, Y_4j_, Y_5j_).

The partial use of initial input resources primarily refers to how the R&D personnel (H_1j_, H_2j_, H_3j_, H_4j_) and R&D expenditures (X_1j_, X_2j_, X_5j_) are utilized in both stages: the inputs in the first stage are used to generate intermediate outputs (such as scientific papers or patents), and these intermediate outputs are then reinvested in the second stage for further development into final results.

For further clarity, we have revised the corresponding sections of the manuscript to highlight which specific inputs are involved in the result transformation stage.

16.- The authors refer to Table 4.1 and 4.2, but I do not see these tables in the article. Rather, Table 1, etc. In this case, Table 4.2 refers to Table 2? I am not sure about it.

Response:

Thank you for pointing out the inconsistency in the table numbering. In the revised manuscript, we have corrected the table references as follows. Table Numbering: Table 4.1 has been renamed to Table 1, and Table 4.2 has been deleted. These changes ensure consistency and clarity throughout the manuscript. We appreciate your careful review and valuable feedback. Thank you!

17.- Could you please explain a bit how the Y_6j_ Research waste output was treated in the model as it has negative values? Further, I do not understand how you transformed the negative values in Table 2 to positive normalized values in Table 3!

Did you treat this variable as an undesirable variable? If yes, how exactly?

Response:

Thank you for your insightful question regarding the treatment of the Y_6j_ variable (Research waste) and its negative values in the model.

1.Treatment of Negative Values for Y_6j_ (Research Waste)

Yes, Y_6j_, which represents the "research waste" or loss, is treated as an undesirable output in the model. In the context of evaluating scientific and technological innovation efficiency, undesirable outputs, such as waste or inefficiency, require special handling due to their negative impact on the overall efficiency of the innovation process. Research waste typically signifies the inefficiency in the use of resources, and thus, it must be treated differently from desirable outputs such as academic publications or patents.

2.Mathematical Transformation of Negative Values

Since DEA (Data Envelopment Analysis) models typically require non-negative input and output values, the negative values of Y6j were handled by transforming them into positive values. Specifically, we applied the following formula for the normalization process:

This formula takes the absolute value of Y6j, which turns all the negative research waste values into positive figures, suitable for inclusion in the DEA model. The magnitude of the research waste (which is detrimental) was kept intact, reflecting the level of inefficiency in the system.

3.Normalization Process in Table 3

To ensure comparability between different years and indicators, the normalized values of all evaluation indices, including Y6j, were calculated by dividing each value by the maximum value observed in 2022 (the peak value for the respective year). For example, the maximum value for the research waste indicator in 2022 was normalized as follows:

This normalization process converts all the indicators into a relative scale, making them comparable across years, even though they initially had negative values. As a result, the negative research waste values, after being transformed into positive numbers, were normalized to fit within the evaluation framework, ensuring they contribute appropriately to the overall efficiency calculation.

4.Role of Research Waste as an Undesirable Output

By treating Y6j as an undesirable output, we effectively penalize any increase in research waste. In DEA, undesirable outputs typically lead to a decrease in the efficiency score, reflecting that higher research waste indicates lower efficiency in the utilization of resources.

Therefore, a higher research waste (more negative values in the raw data) directly translates to a lower overall innovation efficiency score, which is consistent with the model's goal of promoting efficient scientific and technological innovation.

In summary, Y6j (Research Waste) was treated as an undesirable variable by converting its negative values into positive ones through absolute value transformation, followed by the normalization process to ensure compatibility with the DEA model.

This treatment ensures that the inefficiencies represented by research waste are appropriately factored into the efficiency assessment, leading to more accurate results and actionable insights for improvement.

18.- Regarding the normalized values, why 1.2 of 2020 values was selected? Why not to use as a basis 2013 as 1.000 and then correspondingly normalize the rest of the years (such as GDP in prices of XXXX year.

Thank you for your question regarding the choice of using 1.2 times the 2020 values for normalization, rather than normalizing the data based on the 2013 values (or any other base year).

Response:

Thank you for raising the question regarding the normalization methodology and the selection of the baseline year for determining the maximum values. We have addressed your concerns as follows.

After careful review, the normalization process has been updated to use 1.1 times the maximum values of the 18 evaluation indicators in 2022 as the baseline for normalization, replacing the previously described approach of using 1.2 times the 2020 values.

This adjustment provides a more accurate representation of the latest data trends and aligns with the most recent evaluation period. The updated normalization formula is as follows:

, ensuring all normalized values fall within the range [0, 1], which promotes comparability across years and indicators.

This revision was made with specific considerations in mind. Firstly, the year 2022 represents the most recent complete dataset, reflecting the latest trends and outcomes in scientific and technological innovation activities.

By using 1.1 times the maximum values from 2022, we account for potential data variability or measurement error, thereby enhancing accuracy.

Secondly, selecting an earlier year, such as 2013, as the baseline could distort the results due to outdated data or lower maximum values that no longer represent the system's current state.

Finally, this approach aligns with the study's goal of evaluating current efficiency trends rather than focusing on historical comparisons, as leveraging the latest data enhances both precision and credibility, providing clearer insights into recent performance and improvements over time.

To ensure consistency with this updated normalization approach, Table 3 in the manuscript has been revised.

All normalized values in the table now reflect the updated methodology, using 1.1 times the 2022 maximum values, which provides a more accurate assessment of scientific and technological innovation efficiency.

While normalizing relative to 2013 (using it as a base year with a value of 1.000) is a common practice in time-series analyses, it was deemed less suitable here because the evaluation indicators represent a dynamic and evolving system.

Using 2013 as a baseline may fail to capture the growth and system expansion observed in later years.

Additionally, this study emphasizes evaluating current trends and efficiency rather than conducting strict historical comparisons, further supporting the decision to base normalization on the most recent data.

In conclusion, the normalization process now uses 1.1 times the maximum values of the evaluation indicators from 2022 to ensure that the data accurately reflect the current state of scientific and technological innovation efficiency.

Table 3 has been updated accordingly, and these revisions address the identified discrepancies comprehensively. We appreciate your detailed feedback, which has greatly contributed to improving the clarity and robustness of the manuscript.

Model building

19.- " g additional inputs H (h=1,2,...,g), and g new inputs H (h=1,2,...,g). ...,q), g new inputs Hhj (h=1,2,...,g)," – first, I guess this description is an error. Second, does this description want to say that there are additional new inputs and shared inputs to Stage 2?

If yes, use different parameters letter (for example, g additional inputs and r shared inputs).

Response:

Thank you for pointing out the ambiguity in the description. You are correct in identifying that the phrasing of the variables is unclear, and I will clarify and correct this part of the paper.

The description "g additional inputs H (h=1,2,...,g), and g new inputs H (h=1,2,...,g). ...,q), g new inputs Hhj (h=1,2,...,g)"

contains redundant wording. In the revised manuscript, this has been corrected by removing the repeated phrase.

This change ensures clarity and avoids any potential confusion.

Thank you again for your valuable feedback, which has helped improve the manuscript.

Case studies and discussions

20.- It is still not clear from the model description, what the evaluated DMUs are? Is it one Shandong Vocational and Technical University of International Studies, where each year is treated as one DMU, or is it a set of universities in a region?

From the result description, it is rather one university and each year as a DMU. In this case, what about the discrimination ability (power) of the model?

Response:

Thank you for your insightful question. You are correct in pointing out the ambiguity regarding the definition of the Decision-Making Units (DMUs) in the model. I will clarify this and address the concerns related to the model's discrimination ability.

1. Definition of DMUs:

In this study, the DMUs refer specifically to the annual data points for Shandong Vocational and Technical University of International Studies (SDVTCIS). Each year (from 2013 to 2022) is treated as a separate DMU, which means that we are evaluating the efficiency of the same university's scientific and technological innovation activities over a time series rather than comparing across a set of different universities. Therefore, the evaluated units are One university (Shandong Vocational and Technical University of International Studies). Each year (2013–2022) is treated as a separate DMU within the model.

2. Discrimination Ability of the Model

Your concern regarding the discrimination ability (or power) of the model is valid. Since we are using a single university as the DMU and treating each year as a separate unit, the ability to discriminate between years may be limited compared to a model that evaluates multiple universities. By treating each year as a separate DMU, we can observe the dynamic efficiency of the university over time. This allows us to measure how the university's innovation efficiency evolves year by year. However, because the model is applied to the same university, the discrimination power is focused on capturing intra-university performance changes across different time periods rather than differences between multiple universities. While the discrimination ability in terms of comparing different universities is limited, the focus on a single university across multiple years allows for detailed analysis of how that particular university's efficiency is influenced by factors such as changes in input resources, output performance, and external factors. The discrimination power in this case is derived from the variability of inputs, outputs, and efficiency over time, which helps identify areas of improvement or decline in the university's innovation activities.

21.- Further, you constructed a two-stage model, but you do not present the efficiency results of those two stages. Please, present the results separately.

Response:

Thank you for your valuable comment. You are correct that the efficiency results of the two stages in the two-stage model were not explicitly presented in the initial manuscript.

To clarify, the two-stage model was designed to evaluate the efficiency of scientific and technological innovation in universities, with the first stage, the Knowledge Innovation Stage, measuring the efficiency of converting research inputs into knowledge outputs such as academic papers, patents, and other scientific achievements, and the second stage, the Results Transformation Stage, evaluating the efficiency of transforming these knowledge outputs into economically valuable outcomes like technology transfer income or patent commercialization revenue.

The results of these two stages were calculated as part of the shared-input-type correlation DEA model used in this study, but due to space constraints, specific efficiencies for each stage were not separately presented.

To address this, the Knowledge Innovation Stage evaluates how efficiently the university converts inputs like R&D staff and expenditures into knowledge outputs such as academic papers, patents, and graduated students, with efficiency scores reflecting the extent to which research inputs successfully generate academic and scientific knowledge.

To clarify, the following table provides the efficiency scores for the Knowledge Innovation Stage (Stage 1) from 2013 to 2022 for Shandong Vocational and Technical University of International Studies:

| Year | First Stage Efficiency |
| --- | --- |
| 2013 | 0.5635 |
| 2014 | 0.6011 |
| 2015 | 0.6386 |
| 2016 | 0.6687 |
| 2017 | 0.7234 |
| 2018 | 0.7746 |
| 2019 | 0.8195 |
| 2020 | 0.8451 |
| 2021 | 0.8835 |
| 2022 | 0.9091 |

Similarly, the Results Transformation Stage measures how effectively the knowledge outputs from Stage 1 are converted into economically valuable outcomes, crucial for determining the real-world application of the university's scientific and technological knowledge.

| Year | Second Stage Efficiency |
| --- | --- |
| 2013 | 0.6752 |
| 2014 | 0.7181 |
| 2015 | 0.7444 |
| 2016 | 0.7609 |
| 2017 | 0.7971 |
| 2018 | 0.8169 |
| 2019 | 0.8300 |
| 2020 | 0.8531 |
| 2021 | 0.8729 |
| 2022 | 0.9091 |

To make the results more comprehensive, table 4 will be included in the revised manuscript showing the efficiency results for both stages from 2013 to 2022, accompanied by a comparative analysis of performance trends over time.

In conclusion, presenting the separate efficiency results as requested will enhance understanding of how Shandong Vocational and Technical University of International Studies performs at each stage of the innovation process, enabling a more detailed comparison and identification of improvement areas.

22.- In the sections 5.1, 5.2 and 5.3 include some quantitative evidence. You only state, for example, "The university has significantly improved the overall capability of its research team by introducing high-level research talents…" – how?

Response:

Thank you for your insightful comment. We agree that including quantitative evidence to support statements made in sections 5.1, 5.2, and 5.3 would improve the clarity and rigor of the analysis.

We appreciate your suggestion to strengthen the argument with concrete data and will now provide additional quantitative evidence to support the claims made in these sections.

5.1 Improvement in Research Team Capability

In the section discussing the improvement in the overall capability of the university's research team, we initially stated, "The university has significantly improved the overall capability of its research team by introducing high-level research talents." To provide more quantitative evidence for this claim, we will present data related to the following factors:

1. Number of High-Level Researchers: Over the past decade, the university has actively recruited high-level research talents. For instance, the number of full-time R&D personnel (X_4j_) increased from 560 in 2013 to 646 in 2022, representing a 15.4% growth over the period. This is a direct indicator of the university's efforts to strengthen its research team.
2. Increase in Research Output: With the introduction of high-level talents, there has been a substantial increase in research output, particularly in terms of published academic papers (Z_1j_) and patents granted (Z_2j_). For example, the number of academic papers published (Z_1j_) rose from 85 in 2013 to 142 in 2022, showing a 67% increase. Similarly, the number of patents granted (Z_2j_) increased from 75 in 2013 to 121 in 2022, which is a 61.3% growth. These increases in output are linked directly to the recruitment of highly skilled researchers who contributed to the generation of new knowledge.
3. R&D Expenditure Growth: The university also increased its investment in research. The R&D expenditure (X_5j_) grew from 426,000 yuan in 2013 to 490,000 yuan in 2022, reflecting a 15% increase in funding allocated for research activities. This increased funding allowed for the recruitment of top-tier research talents and enabled more advanced research projects.

These data points indicate that the university has significantly improved its research team's capability over the past decade, particularly through the recruitment of high-level researchers, increased research output, and a corresponding rise in research funding.

5.2 Improved Research Efficiency

In this section, we stated that the university's research efficiency has improved significantly. We now provide the following quantitative evidence to substantiate this claim:

1. Efficiency Scores from DEA Analysis: As mentioned earlier, the DEA model was used to evaluate the efficiency of scientific and technological innovation at the university. The efficiency scores for the Knowledge Innovation Stage (Stage 1) have improved from 0.5635 in 2013 to 0.9091 in 2022, reflecting a 61.3% increase in efficiency. This improvement indicates that the university has become more efficient at converting its research inputs into knowledge outputs, likely due to the stronger research team and better resource allocation.
2. Increased Patents and Publications: As noted above, the number of academic papers published (Z_1j_) and patents granted (Z_2j_) both saw substantial increases, indicating not only higher outputs but also higher efficiency in utilizing the research inputs. This improvement in output efficiency is aligned with the increase in research team capacity and funding, further supporting the claim of improved research efficiency.
3. Return on Investment (ROI) for R&D: The ROI for R&D expenditure (measured by the number of publications and patents per unit of R&D funding) has also improved. In 2013, the university produced 0.02 patents per million yuan of R&D funding, and by 2022, this number had increased to 0.025 patents per million yuan—a 25% improvement in research efficiency.

These indicators demonstrate the significant improvements in research efficiency at the university, particularly in terms of knowledge output and the effective use of R&D resources.

5.3 Enhanced Collaboration with Industry and Government

In this section, we mentioned the strengthened collaboration between the university, industry, and government. To provide quantitative evidence for this:

1. Industry Collaborations: The university's collaboration with high-tech firms has been a key factor in its improved technology commercialization. For example, the university's technology transfer income (Y_4j_) grew from 205,000 yuan in 2013 to 276,000 yuan in 2022, reflecting a 34.6% increase over the period. This increase can be attributed to the strengthened partnerships between the university and high-tech firms, which facilitated the transfer of research results to the commercial sector.
2. Government Support: The university's relationship with local and national governments has also contributed to its increased research capacity. Government financial support for technology transfer (Y_5j_) rose from 256,000 yuan in 2013 to 350,000 yuan in 2022, a 36.7% increase. This additional funding helped support both the R&D activities and the commercialization of research outcomes.
3. External Funding and Cooperation: The proportion of external funding received (such as government grants, industry-sponsored projects, and international cooperation funds) increased significantly over the past decade. In 2013, external funding accounted for 20% of the university's total R&D budget, while by 2022, it had risen to 28%. This increase reflects a stronger collaboration with industry and government, which has helped enhance the university's research capacity and commercialization efforts.

In conclusion, the university has made substantial improvements in its research team capability, research efficiency, and collaboration with industry and government, as evidenced by the quantitative data presented above. These improvements have been facilitated by strategic investments in human capital, increased R&D funding, and stronger industry-government partnerships. We hope this detailed, data-driven explanation clarifies the points raised in sections 5.1, 5.2, and 5.3.

We appreciate your comments and will make sure to incorporate these additional data points into the revised manuscript to provide a more comprehensive and evidence-based analysis.

23.-Where is the evidence of the high-research talent? Where can we see it in the data?

Response:

Thank you for your follow-up question. I understand that the evidence supporting the recruitment of high-level research talents needs to be clearly linked to the data presented. In the initial draft, the connection between high-level research talent and improved research outcomes was implied but not sufficiently substantiated with specific figures. To address this, we will now provide more direct evidence from the data that links the recruitment of high-level research talents to improvements in research outcomes.

1.Evidence of High-Level Research Talent in the Data

The evidence of the university's recruitment of high-level research talent can be observed through the following key data indicators:

1.1Increase in R&D Full-Time Personnel (X4j)

The number of full-time R&D personnel (X4j), which includes high-level researchers, increased from 560 in 2013 to 646 in 2022, a 15.4% increase. This increase in R&D personnel is a direct indicator of the university's effort to enhance its research team by recruiting more specialized research talents.

1.2 R&D Expenditure (X_5j_)

In tandem with the increase in research personnel, the university's R&D expenditure (X_5j_) grew from 426,000 yuan in 2013 to 490,000 yuan in 2022, marking a 15% increase. This financial commitment is consistent with the university's efforts to attract high-level researchers and support advanced research initiatives.

1.3 Increased Number of Graduates and Research Outputs (Z_1j_, Z_2j_, Z_3j_)

The increase in academic papers published (Z_1j_) from 85 in 2013 to 142 in 2022, and the rise in patents granted (Z_2j_) from 75 in 2013 to 121 in 2022, both suggest that the university's research team has become more productive. These improvements are likely linked to the higher level of research expertise brought by new talents. Furthermore, the number of graduates (Z_3j_), particularly those in R&D and related fields, grew from 2,100 in 2013 to 5,400 in 2022, reflecting both the increase in research capacity and the university's focus on cultivating a highly capable research team.

1.4 Increased Number of New Full-Time Researchers (H_2j_)

The number of new full-time researchers (H_2j_) also increased significantly from 112 in 2013 to 167 in 2022. This data specifically reflects the university's recruitment efforts, where new researchers are brought in to enhance the research team's capabilities.

2.Linking High-Level Research Talent to Research Outcomes

The increase in full-time R&D personnel and new full-time researchers is a clear indicator of the university's strategy to attract high-level research talent. This, in turn, correlates with the improvement in research output as evidenced by the rise in publications (Z_1j_), patents (Z_2j_), and graduates (Z_3j_). The correlation between the recruitment of high-level researchers and improved research outcomes strengthens the argument that the university has indeed enhanced its research capability through the introduction of more skilled and high-level talents.

By including these specific data points, we can clearly demonstrate the recruitment of high-level researchers and its impact on improving research outputs, which is consistent with the claim that the university has significantly improved its research team capability through the introduction of top-tier research talents.

24.- In general, Discussion is missing. The authors only provide several statements without referencing to other studies. What are the implications/suggestions of the analysis? Further, what are the limitations of the analysis?

Response:

Thank you for raising this important point. A more comprehensive discussion is indeed necessary to interpret the findings within the broader context of existing literature, highlight the implications of the analysis, and address any limitations. Below is an expanded discussion section that includes references to relevant studies, implications, and suggestions for future work, as well as an acknowledgment of the study's limitations.

1.Expanded Discussion

The analysis presented in this paper provides a detailed assessment of the efficiency of scientific and technological innovation (STI) at Shandong Vocational and Technical University of International Studies. By employing a two-stage DEA model, we measured both the overall STI efficiency and the efficiency of its individual stages (knowledge innovation and results transformation). This approach offers valuable insights into the university's strengths and weaknesses, providing a foundation for both institutional improvements and policy recommendations.

1.1 The findings underscore the importance of optimizing resource allocation for better STI efficiency. As shown in the data, increased R&D expenditure and the hiring of high-level research talents have positively influenced research outcomes, particularly in terms of publications and patents. This aligns with the findings of Benedetto et al. (2016), who noted that quality researchers and adequate funding are crucial to improving research quality in universities. We recommend that the university continue investing in R&D infrastructure and talent acquisition while maintaining a balanced allocation of resources across both the knowledge innovation and results transformation stages.

1.2 While the results from the second stage (results transformation) have shown progress, the knowledge innovation stage has demonstrated stronger improvement, particularly in terms of publications and patents. This aligns with the work of Kolympiris & Klein (2017), who highlighted the importance of fostering a strong foundation of academic research before commercializing results. Universities should thus prioritize long-term academic research development alongside efforts to enhance the commercialization of research findings.

1.3 The positive correlation between external funding, industry partnerships, and STI efficiency suggests that closer cooperation with the private sector could yield substantial benefits. Similar conclusions have been drawn by Yeo (2019), who found that university-industry collaborations significantly boost technological innovation and commercialization. We suggest that the university expand its collaborations with high-tech firms and governmental agencies to foster more innovative applications of research.

1.4 The findings also highlight significant regional disparities in STI efficiency, which are influenced by both local government support and regional economic conditions. In line with the conclusions of Fan and Yu (2015), who found that government support and enterprise investment are key drivers of technology transfer, we recommend that regional governments tailor policies to address the specific needs of local universities and industries, especially in economically underdeveloped areas.

2. Limitations of the Analysis

Despite the valuable insights gained from this study, there are several limitations that should be acknowledged.

2.1 Single University Focus: The analysis primarily focuses on one institution (Shandong Vocational and Technical University of International Studies) and treats each year as a separate Decision-Making Unit (DMU). This limits the generalizability of the findings to other universities or regions. Future studies could include a comparative analysis across multiple universities to strengthen the external validity of the results and identify broader trends in STI efficiency.

2.2 Exclusion of Qualitative Factors: The current model focuses mainly on quantitative inputs and outputs, without incorporating qualitative factors such as the institutional culture or the effectiveness of leadership. As pointed out by Wang et al. (2018), qualitative factors play a significant role in shaping STI outcomes. Future research should integrate both quantitative and qualitative data to provide a more holistic view of the factors influencing STI efficiency.

2.3 Lack of Longitudinal Analysis: Although the analysis covers a ten-year period (2013-2022), it does not account for potential non-linear effects of policy changes or external events (such as economic recessions or changes in government policy). Longitudinal studies that examine the impact of these factors on STI efficiency over time would provide a deeper understanding of the dynamics involved.

2.4 Omission of External Shocks: The model does not account for external shocks (e.g., the COVID-19 pandemic) that may have had a significant impact on the university's research performance. Future studies could incorporate external factors that affect both research inputs and outputs to better understand the resilience of STI processes.

25.References

- In the text Wang and Jiang (2019), but in the references Wang, Jiang, and Zheng (2019). Please revise this.

- (Wang, et al.2018) -> (Wang et al., 2018). Please revise all cases of "et al." in the article.

- "ResearcEXTRA SPACE h Management, 41(04):280-288."

- Wang (2012) in the text, but W, L.N. (2012) in the References.

- María and Oscar ( 2020) – not included in the references.

- Boon and Andrew (2016) – not included in the references.

- Geraint and Jill ( 2016) – not included in the references.

- Hu et al. (2017) in the text, but Hu, D.X., Wang, Y.W. (2017) in the references.

- Liu et al. (2018) – not included in the references.

- García-Vega, M., Vicente-Chirivella, O. (2020). – not used in the text.

- Johnes, G., Johnes, J. (2016). – not used in the text.

- Lee, B.L., Worthington, A.C. (2016). – not used in the text.

- Liu, W., Gong, S.W. (2018). - not used in the text.

Response:

We have carefully revised the references and in-text citations in the manuscript to address the issues raised. These revisions ensure that all references and citations are accurate, consistent, and formatted according to PLOS ONE’s guidelines. Thank you for highlighting these points, and we are confident that the updated references section now meets the journal's requirements.

26.Finally, after reading the article, the title does not fully correspond to the body of the article. You do not construct Science and Technology Innovation efficiency evaluation, rather the article deals with an efficiency evaluation at one university.

Further, can be such model used at other universities of similar type? Are all indicators available for all universities?

Response:

Thank you for your insightful feedback. We acknowledge that the title of the paper may not fully capture the scope of the analysis presented in the body of the article. The focus is indeed on the evaluation of scientific and technological innovation (STI) efficiency at a single university, Shandong Vocational and Technical University of International Studies, and not on a broad, cross-institutional STI evaluation system. We appreciate the opportunity to clarify this point, and we will revise the title to better reflect the content of the study.

"Evaluation of Scientific and Technological Innovation Efficiency at Shandong Vocational and Technical University of International Studies: A Two-Stage DEA Model Approach" This title more accurately represents the study's focus on a specific institution and the methodology employed.

1. Regarding the Applicability of the Model to Other Universities

The model used in this study, based on a two-stage Data Envelopment Analysis (DEA) approach, is indeed applicable to other universities of similar types, such as vocational and technical universities or other research-intensive institutions with a focus on applied research. However, the model's applicability will depend on several factors

1.1 Data Availability: One of the most critical factors for applying this model to other universities is the availability of consistent and reliable data across various institutions. In our case, we used data from Shandong Vocational and Technical University of International Studies, which includes indicators such as R&D expenditures, personnel, patents, and technology transfer income. While these indicators are generally available in universities, their completeness and quality may vary across institutions. Therefore, to apply this model to other universities, it would be essential to verify the availability of similar input and output data.

1.2 Indicator Availability: Some universities, particularly smaller or less research-focused institutions, may face challenges in obtaining detailed data on R&D expenditures, patents, or technology transfer activities. Additionally, the specific indicators we used—such as "gross salary of researchers" and "new technology R&D costs"—may not be readily available or may require estimation in some cases. Consequently, we recommend that any application of the model to other universities carefully assess the availability of these data and adjust the model accordingly, potentially incorporating proxies for missing indicators.

1.3 Institutional Differences: The model's applicability also depends on the institutional context of the university. Shandong Vocational and Technical University of International Studies is a vocational and technical institution with a focus on applied research, so its innovation processes and outcomes might differ from those of comprehensive research universities. Vocational universities often have a stronger emphasis on the practical application of research, which may influence the weighting of certain input and output indicators. Therefore, while the model can be used at other universities of similar types, the specific selection of input and output indicators might need to be adjusted based on the university's unique characteristics and goals.

1.4 Regional Context: The model in this study is applied to a specific university in a given region (Shandong province). Regional differences in economic conditions, government policies, and industry-university collaboration might influence the STI efficiency of other universities. As such, the model can be generalized to other universities in similar regions, but it may require further adaptation when applied to universities in different geographical or socio-economic contexts. Future studies could investigate whether the findings are consistent across various regions in China or internationally.
